# A Newly Reported European Epidemic

**Published:** 1890-05

**Authors:** 


### A NEWLY REPORTED EUROPEAN EPIDEMIC.

---

According to cablegrams from the continent a new plague has sprung up, called nouna or noma, in Russia, Austria and Italy. It bears no resemblance to any malady of recent times. The marked feature of the attack is a stupor or prolonged sleep, of twenty-four to forty-eight hours' duration. This may come on suddenly in the midst of apparent good health, or may be preceded by two or three days of insomnia, headache and malaise. Fatal cases have occurred, the patient never awakening, or the stupor passes off and recovery follows. Whether the disease is cantagious, or otherwise, is not yet known.—*St. Louis Med. and Surg. Journal.*

---

Any one interested in the sick-benefit, funeral-aid, and death beneficiary associations of the United States can help make the statistics of their organizations for the forthcoming census more complete and disseminate the knowledge of the good work they are doing by sending the names of such societies as they may know of, and the addresses of their principal officers, to Mr. Charles A. Jenney, Special Agent of the Eleventh Census, 58 William street, New York City.

